# Omecamtiv mecarbil evokes diastolic dysfunction and leads to periodic electromechanical alternans

**DOI:** 10.1007/s00395-021-00866-8

**Published:** 2021-04-12

**Authors:** Gábor Á. Fülöp, Attila Oláh, Tamas Csipo, Árpád Kovács, Róbert Pórszász, Roland Veress, Balázs Horváth, László Nagy, Beáta Bódi, Miklós Fagyas, Solveig Lind Helgadottir, Viktor Bánhegyi, Béla Juhász, Mariann Bombicz, Daniel Priksz, Peter Nanasi, Béla Merkely, István Édes, Zoltán Csanádi, Zoltán Papp, Tamás Radovits, Attila Tóth

**Affiliations:** 1 Division of Clinical Physiology, Department of Cardiology, Faculty of Medicine, University of Debrecen, 22 Móricz Zsigmond Street, 4032 Debrecen, Hungary; 2 Doctoral School of Kálmán Laki, University of Debrecen, Debrecen, Hungary; 3 Heart and Vascular Center, Semmelweis University, Budapest, Hungary; 4 Division of Cardiology, Department of Cardiology, Faculty of Medicine, University of Debrecen, Debrecen, Hungary; 5 Department of Pharmacology and Pharmacotherapy, Faculty of Medicine, University of Debrecen, Debrecen, Hungary; 6 Department of Physiology, Faculty of Medicine, University of Debrecen, Debrecen, Hungary; 7 Department of Biophysics and Cell Biology, Faculty of Medicine, University of Debrecen, Debrecen, Hungary; 8 HAS-UD Vascular Biology and Myocardial Pathophysiology Research Group, Hungarian Academy of Sciences, Budapest, Hungary

**Keywords:** Omecamtiv mecarbil, Inotropy, Diastolic dysfunction, *Pulsus alternans*, Heart failure, Ca^2+^ sensitivity

## Abstract

Omecamtiv mecarbil (OM) is a promising novel drug for improving cardiac contractility. We tested the therapeutic range of OM and identified previously unrecognized side effects. The Ca^2+^ sensitivity of isometric force production (pCa_50_) and force at low Ca^2+^ levels increased with OM concentration in human permeabilized cardiomyocytes. OM (1 µM) slowed the kinetics of contractions and relaxations and evoked an oscillation between normal and reduced intracellular Ca^2+^ transients, action potential lengths and contractions in isolated canine cardiomyocytes. Echocardiographic studies and left ventricular pressure–volume analyses demonstrated concentration-dependent improvements in cardiac systolic function at OM concentrations of 600–1200 µg/kg in rats. Administration of OM at a concentration of 1200 µg/kg was associated with hypotension, while doses of 600–1200 µg/kg were associated with the following aspects of diastolic dysfunction: decreases in E/A ratio and the maximal rate of diastolic pressure decrement (dP/dt_min_) and increases in isovolumic relaxation time, left atrial diameter, the isovolumic relaxation constant Tau, left ventricular end-diastolic pressure and the slope of the end-diastolic pressure–volume relationship. Moreover, OM 1200 µg/kg frequently evoked transient electromechanical alternans in the rat in vivo in which normal systoles were followed by smaller contractions (and T-wave amplitudes) without major differences on the QRS complexes. Besides improving systolic function, OM evoked diastolic dysfunction and *pulsus alternans*. The narrow therapeutic window for OM may necessitate the monitoring of additional clinical safety parameters in clinical application.

**Supplementary Information:**

The online version contains supplementary material available at 10.1007/s00395-021-00866-8.

## Introduction

Omecamtiv mecarbil (OM) is a promising drug candidate to improve cardiac contractility (inotropy) by selectively activating cardiac myosin [11, 25]. In a clinical dose-ranging study, it prolonged systolic time, increased stroke volume, decreased left ventricular dimension and reduced heart rate [3]. It has also been reported to cause a decrease in levels of the heart failure biomarker N-terminal pro-brain natriuretic peptide [27]. The proposed molecular mechanism of the actin–myosin cycle [11, 16, 18, 21] suggests that OM selectively targets myosin, a key molecule in cardiac contraction, without the involvement of additional molecular mechanisms. However, a recent report challenged this view of the apparent selectivity of OM and put forward a completely new hypothesis in which the selective suppression of myosin was associated with cooperative thin-filament activation [31]. There are also some recent reports suggesting that OM evokes a rise in myocardial oxygen consumption [1], and affects intracellular Ca^2+^ homeostasis [15] and repolarization of cardiomyocytes [24] at higher concentrations (3–10 µM). Moreover, in vitro, OM not only prolongs cardiomyocyte contraction but also slows down relaxation [12, 14].

In this study, we aimed to test the cellular effects of OM in permeabilized human left ventricular cardiomyocytes and intact canine cardiomyocytes in vitro, and to study the in vivo effects in the rat. We identified previously unrecognized effects and found a narrow therapeutic window for OM. In particular, diastolic dysfunction was detected in parallel with positive inotropy, while severe hypotension and periodic electromechanical and T-wave alternans developed as the dosage of OM increased.

## Methods

### Human tissue source

Human tissue was obtained as described previously [13]. Healthy human hearts were obtained from five general organ donor patients (two men and three women with a mean age of 39.2 years) whose hearts were explanted. The experiments complied with the Helsinki Declaration of the World Medical Association at the time of the study and were approved by the Hungarian Ministry of Health (approval number 323-8/2005-1018EKU). Left ventricular (LV) wall samples were frozen in liquid nitrogen and stored at − 80 °C.

### Animal experiments

All animal care and experimental procedures conformed to Directive 2010/63/EU of the European Parliament and were approved by the appropriate ethical committees (approval number 1/2013/DE MAB). In vivo experiments were carried out on 13–15-week-old male Wistar–Kyoto rats. Myocytes for action potential (AP), cell shortening and intracellular calcium measurements were isolated from 12–18-month-old mongrel dogs.

### Hemodynamic measurements

In vivo hemodynamic measurements were performed according to a previously described method [17], with specific modifications for this study, as detailed in the supplementary material.

### Echocardiography

Effects of OM were tested in rats echocardiographically using a General Electric Vivid E9 ultrasound system equipped with a linear 14.1-MHz i13L probe (General Electric, Fairfield, CT) as described previously [7], with specific modifications for this study, as detailed in the supplementary material.

### Invasive blood pressure measurement

Effects of OM on blood pressure were tested in rats by cannulating the carotid artery. Arterial pressure and electrocardiographic signals were monitored simultaneously. A detailed description can be found in the supplementary material.

### Mechanical measurements on permeabilized myocytes

Isometric force generation of isolated human single LV cardiomyocytes was measured at sarcomere lengths of 2.3 μm, according to a previously described method [14]. A description of these experiments can be found in the supplementary material.

### *Simultaneous recording of AP, cell length and Ca*^*2*+^*transients in isolated canine cardiomyocytes*

Single cardiomyocytes were obtained from adult mongrel dogs by enzymatic dispersion using the segment-perfusion technique [10]. Experiments were performed as described previously [6], with slight modifications, as detailed in the supplementary material.

### Estimation of short-term variability

A series of 20 consecutive single-cell contractions (isolated cells) or 12 cardiac cycles (pressure–volume [P–V] loops and echocardiography) were analysed to estimate beat-to-beat variations.

### Omecamtiv mecarbil (OM)

OM was purchased from AdooQ BioScience (Irvine, CA, USA). Stock solutions with concentrations of 1 and 10 mM were prepared in dimethyl sulfoxide (DMSO) and stored at 4 °C.

### Statistics

A detailed description of the statistical methods used can be found in the supplementary material.

## Results

### *Omecamtiv mecarbil affects contraction, relaxation, Ca*^*2*+^*sensitivity and stiffness of Triton-X-100-permeabilized human cardiomyocytes *in vitro

**Fig. 1:**
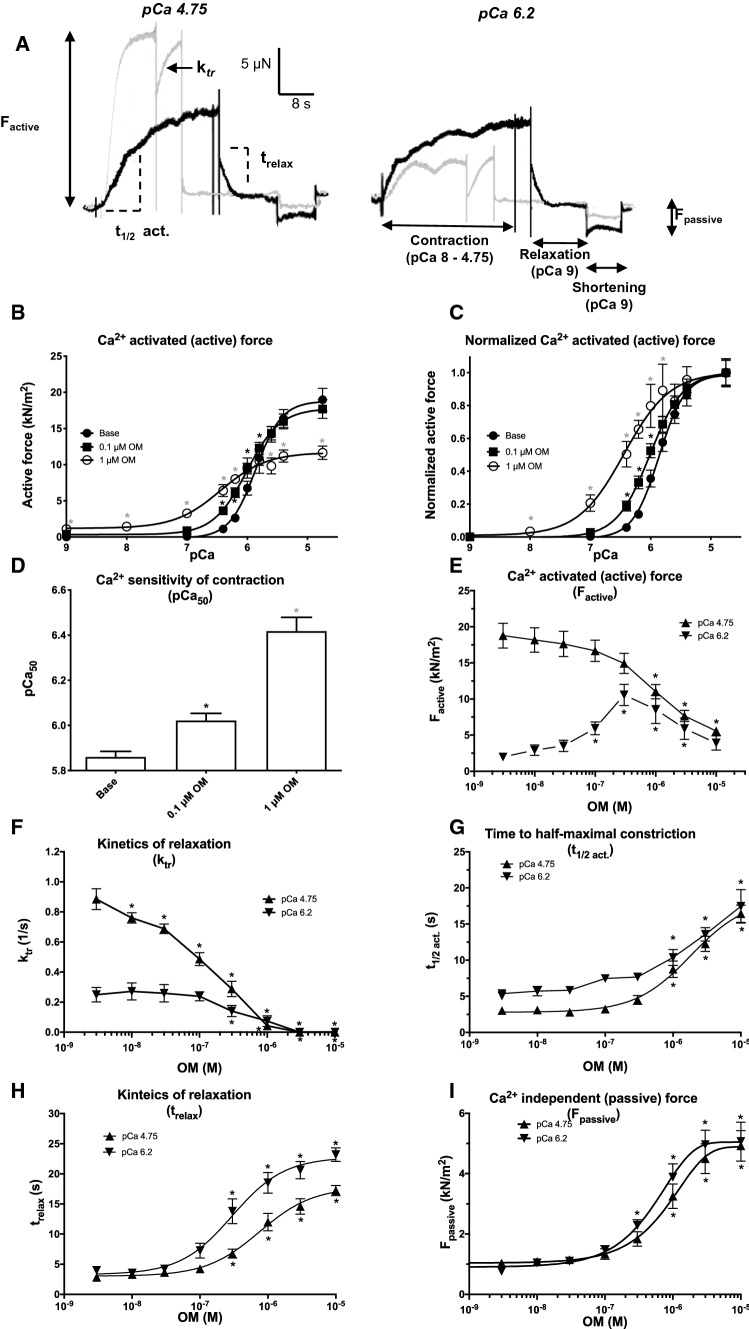
Omecamtiv mecarbil evokes Ca^2+^ sensitization, slower contraction–relaxation kinetics and increased passive stiffness in permeabilized human cardiomyocytes. Original force recordings at maximal (pCa 4.75; ≈18 μM) and submaximal (pCa 6.2; ≈0.63 μM) Ca^2+^ concentrations illustrate the effects of 1 µM omecamtiv mecarbil (OM) on force development in isolated, permeabilized human left ventricular cardiomyocytes. Gray traces: controls; black traces: the same cells with OM (**a**). Steps of the measurement are indicated at pCa 6.2 (black trace). First, the permeabilized ventricular cardiomyocyte was placed in a Ca^2+^-containing solution and the developing Ca^2+^-dependent force was recorded. Then the preparation was transferred to a relaxing solution (pCa 9). After steady-state relaxation, the passive stiffness was determined by shortening the sarcomeres to a slack level. The Ca^2+^ concentration–active force relationship was determined (shown in absolute and relative [normalized] units in panels (**b**) and (**c**), respectively) without (baseline) and with 0.1 and 1 µM OM (as indicated). The bar graph (**d**) represents the mean ± standard error of the mean (SEM) of seven to nine individual measurements (a significant difference [*P* < 0.05] from baseline is indicated by an asterisk). OM had a biphasic effect on the active force development as shown by the concentration–response graph at pCa 4.75 (**e**). The overall rate of active force development (k_tr_; **f**) and the time required for half-maximal Ca^2+^-dependent force development (t_1/2 act_; **g**) are shown on the graphs. Finally, the rate of relaxation (t_relax_; H) and the passive (Ca^2+^-independent) stiffness (F_passive_; **i**) were also determined at different OM concentrations. The applied OM concentrations are shown on the horizontal axes. Symbols represent the mean of seven to nine independent determinations. Error bars indicate the SEM

The effects of OM on Triton-X-100-permeabilized human cardiomyocyte contractility were tested (Fig. 1a) in the presence of low (0.1 µM) and high (1 µM, 401 ng/ml) concentrations of OM (Fig. 1b–d). Force development was significantly increased at very low Ca^2+^ levels in the presence of 1 µM OM. For example, the force value in relaxing solution (pCa 9) was 1.1 ± 0.2 kN/m^2^, compared with 0 kN/m^2^ in the absence of OM (Fig. 1b). The Ca^2+^ sensitivity of force production (pCa_50_) increased from 5.86 ± 0.02 to 6.42 ± 0.06 (Fig. 1c and d) in the presence of 1 µM OM. The same dose of OM increased Ca^2+^-activated force production (F_active_) severalfold at low Ca^2+^ concentrations (F_active_ at pCa 6.2 increased from 1.7 ± 0.3 to 8.6 ± 1.9 kN/m^2^; Fig. 1e), while it decreased maximal Ca^2+^-activated force at the maximal level of activation (F_active_ at pCa 4.75 decreased from 20.4 ± 2.0 to 11.0 ± 1.0 kN/m^2^; Fig. 1e). The rate of Ca^2+^-dependent force production (k_tr_) at an OM concentration of 1 µM decreased irrespective of Ca^2+^ concentration (k from 0.97 ± 0.06 to 0.04 ± 0.01 1/s and from 0.28 ± 0.05 to 0.107 ± 0.04 1/s when measured at high and submaximal Ca^2+^ levels, respectively; Fig. 1f). The time required for half-maximal contraction (t_1/2_ act) increased from 5.06 ± 047 to 10.40 ± 1.11 s and from 2.80 ± 0.21 to 8.75 ± 1.14 s at an OM concentration of 1 µM at low and submaximal Ca^2+^ levels, respectively (Fig. 1g). The kinetics of relaxation (t_relax_) slowed down from 2.67 ± 0.38 to 18.51 ± 1.72 s and from 2.74 ± 0.34 to 12.00 ± 1.44 s at an OM concentration of 1 µM at submaximal and high Ca^2+^ levels, respectively (Fig. 1h). Lastly, the Ca^2+^-independent (passive) stiffness (F_passive_) increased from 0.88 ± 0.10 to 3.88 ± 0.44 kN/m^2^ and from 1.03 ± 0.12 to 3.25 ± 0.41 kN/m^2^ at submaximal and high Ca^2+^ levels, respectively, in response to OM 1 µM (Fig. 1i).

### *Omecamtiv mecarbil evokes positive inotropy in the rat *in vivo

**Fig. 2:**
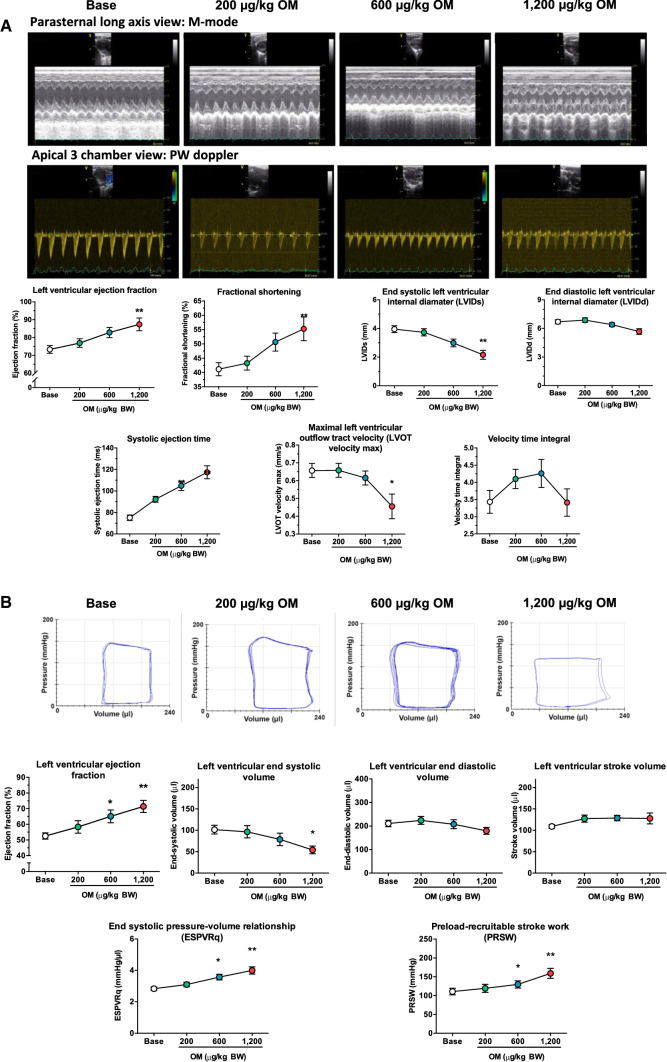
Omecamtiv mecarbil improves left ventricular systolic function in the rat. Omecamtiv mecarbil (OM) was tested in the rat in vivo at cumulative doses of 200–1200 µg/kg body weight. Left ventricular systolic function was studied by echocardiography (**a**) and by left ventricular pressure–volume analysis (**b**). M-mode was used in the parasternal long-axis view (representative recordings shown in the upper row) to determine ejection fraction, fractional shortening, left ventricular end-systolic internal diameter and left ventricular end-diastolic internal diameter. The pulsatile wave (PW) Doppler method (from the left ventricular outflow tract) was used to determine hemodynamic parameters (representative recordings in the lower row of traces), such as left ventricular systolic ejection time, maximal blood flow velocity at the left ventricular outflow tract and left ventricular outflow tract velocity time integral, as shown in the graphs. Representative left ventricular steady-state pressure–volume loops were obtained at different cumulative OM doses during pressure–volume analysis (**b**). Ejection fraction, end-systolic volume, end-diastolic volume, stroke volume and load-independent contractility indices (slope of the end-systolic pressure–volume relationship; preload recruitable stroke work) at different OM doses are shown in the graphs. The number of independent observations was eight to 14 for echocardiography and nine for the pressure–volume analysis. Symbols represent the mean and standard error of the mean. Significant differences from the initial (baseline) values upon application of OM (cumulative doses are shown on the horizontal axes) are indicated by asterisks: *P < 0.5; **P < 0.01

Intravenous, cumulative application of 200, 400 and 600 µg/kg body weight (BW) OM (yielding a maximum dose of 1200 µg/kg BW) improved cardiac systolic function as revealed by echocardiography (Fig. 2a). Ejection fraction (EF) increased from 73.3 ± 2.2 to 87.4 ± 3.6%; fractional shortening (FS) increased from 41.2 ± 2.3 to 55.3 ± 4.1%; LV internal diameter at systole (LVIDs) decreased from 4.0 ± 0.2 to 2.2 ± 0.3 mm; LV internal diameter at diastole (LVIDd) decreased from 6.7 ± 0.2 to 5.7 ± 0.3 mm; and systolic ejection time (SET) increased from 75.2 ± 2.5 to 117.4 ± 6.1 ms. In parallel, maximal blood flow velocity at the left ventricular outflow tract (LVOT velocity) decreased from 0.66 ± 0.04 to 0.46 ± 0.07 mm/s, while the LVOT velocity–time integral did not change significantly.

Improvement in systolic function was also detected by LV P–V measurements under the same conditions (Fig. 2b): EF increased from 52.5 ± 2.0 to 71.4 ± 3.9%. LV end-systolic volume decreased from 101.6 ± 10.2 to 54.2 ± 9.1 µL, while LV end-diastolic volume decreased slightly resulting in a non-significant increase in stroke volume from 109.0 ± 4.5 to 127.8 ± 12.7 µL. Load-independent contractility indices also showed clear benefits in systolic performance: both the end-systolic P–V relationship (ESPVRq) and the preload-recruitable stroke work (PRSW) increased (from 2.8 ± 0.1 to 4.0 ± 0.2 mmHg/µL and from 110.9 ± 8.7 to 159.1 ± 13.2 mmHg, respectively).

### *Omecamtiv mecarbil causes diastolic dysfunction in the rat *in vivo

**Fig. 3:**
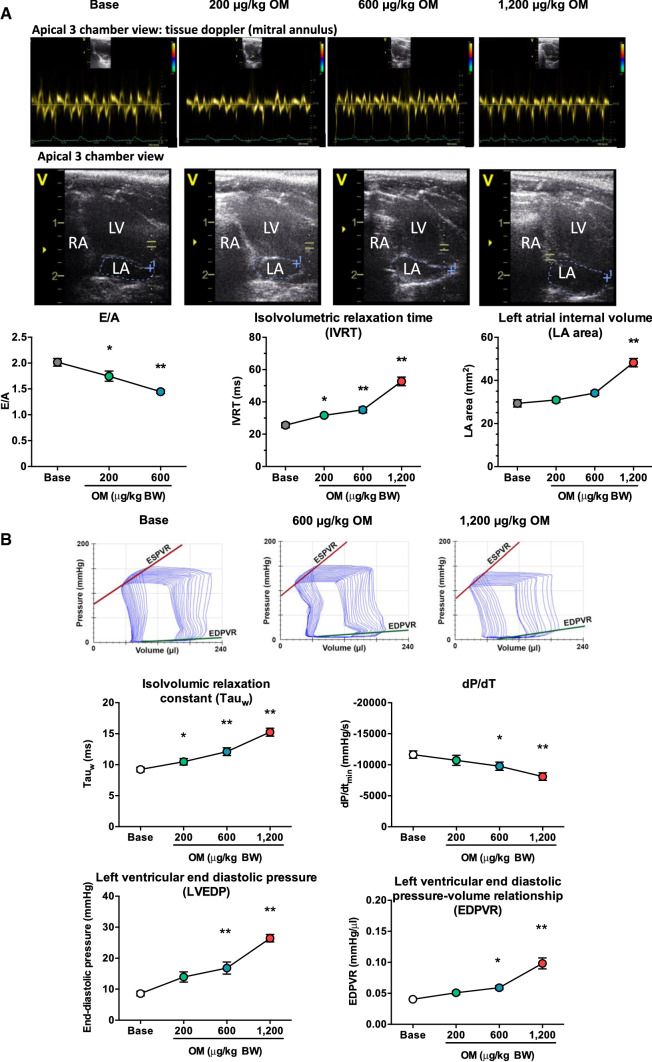
Omecamtiv mecarbil negatively affects left ventricular diastolic filling in the rat. Omecamtiv mecarbil (OM) was tested in the rat in vivo. Left ventricular diastolic function was studied by echocardiography (**a**) and by left ventricular pressure–volume analysis (**b**). Pulsatile-wave Doppler inflow at the mitral valve was used to determine the early:atrial filling ratio (E:A). The tissue Doppler method at the mitral annulus (representative individual recordings in the top row) was used to determine isovolumetric relaxation time. Left atrial area was determined from two-dimensional images in an apical 3 chamber view (representative pictograms are shown in the row below the tissue Doppler recordings) and values are plotted on the graphs. Representative original recordings of left ventricular pressure–volume relationships during transient occlusion of the inferior vena cava at different OM doses are shown in the top row of panel (**b**). The following diastolic parameters were acquired from pressure–volume analysis: indices of left ventricular active relaxation (dP/dt_min_ and the isovolumic relaxation constant) and left ventricular stiffness (end-diastolic pressure and slope of the end-diastolic pressure–volume relationship). The number of independent observations was seven to 14 for echocardiography and nine for the pressure–volume analysis. Symbols represent the mean and standard error of the mean. Significant differences from the initial (baseline) values upon application of OM (cumulative doses are shown on the horizontal axes) are indicated by asterisks: *P < 0.5; **P < 0.01

OM treatment was also associated with signs of diastolic dysfunction on echocardiography (Fig. 3a): peak mitral early and late blood flow velocity ratio (E:A) decreased from 2.02 ± 0.08 to 1.45 ± 0.03; isovolumetric relaxation time (IVRT) increased from 25.6 ± 1.6 to 52.7 ± 2.7 ms and left atrial internal volume (LA area) increased from 29.4 ± 1.7 to 48.3 ± 2.0 mm^2^. Signs of diastolic dysfunction were also present when a P–V analysis was performed (Fig. 3b). The isovolumic relaxation constant (Tau_w_) increased from 9.2 ± 0.4 to 15.2 ± 0.7 ms and the maximal rate of diastolic pressure decrement (dP/dt_min_) decreased from − 11,642 ± 603 to − 8096 ± 614 mmHg/s, indicating impairment of active relaxation. The LV end-diastolic pressure (LVEDP) increased from 8.6 ± 0.8 to 26.4 ± 1.2 mmHg and the end-diastolic P–V relationship (EDPVR) increased from 0.040 ± 0.001 to 0.098 ± 0.009 mmHg/µL, suggesting stiffening of the left ventricle.

### *Omecamtiv mecarbil evokes hypotension in the rat at high dosages *in vivo

**Fig. 4:**
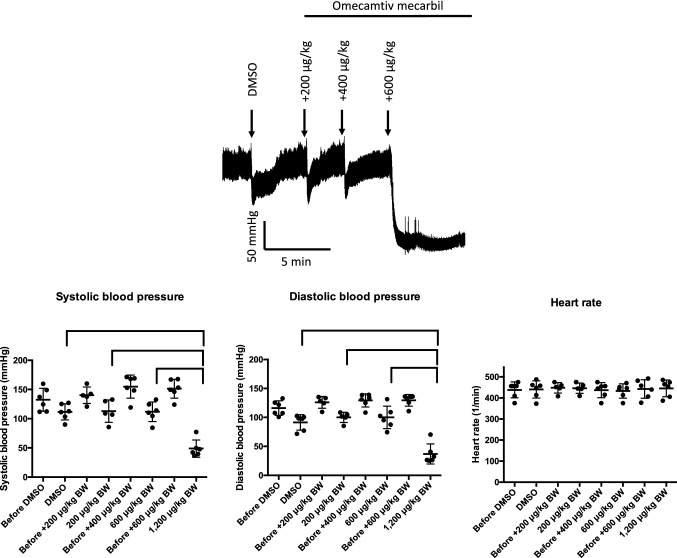
Omecamtiv mecarbil evokes severe hypotension in the rat. Omecamtiv mecarbil (OM) was tested in the rat in vivo. Application of solvent (dimethyl sulfoxide [DMSO]) alone and increasing doses of OM are shown in a representative invasive blood pressure recording at the top. Systolic and diastolic blood pressure and heart rate were determined immediately before and 5 min after bolus injections as indicated on the horizontal axes. Each symbol represents an individual measurement. The mean and standard error of the measurements are shown on the scatter graphs. Significant (*P* < 0.05) differences among the six replicates are indicated by the braces

Invasive blood pressure measurements were performed in rats (Fig. 4). DMSO alone (solvent) led to a slight decrease in blood pressure, although it returned to normal levels within 5 min of application. OM evoked prominent decreases in both systolic (from 149 ± 6 to 49 ± 6 mmHg; *P* < 0.05) and diastolic (from 130 ± 4 to 37 ± 7 mmHg; *P* < 0.05) blood pressure at a cumulative dose of 1200 µg/kg BW. There was no effect on heart rate, irrespective of the development of hypotension (443 ± 18 and 445 ± 16 beats/min before and after treatment, respectively) (Fig. 4).

### *Omecamtiv mecarbil evokes a transient electromechanical alternation in the rat at high dosages *in vivo

**Fig. 5:**
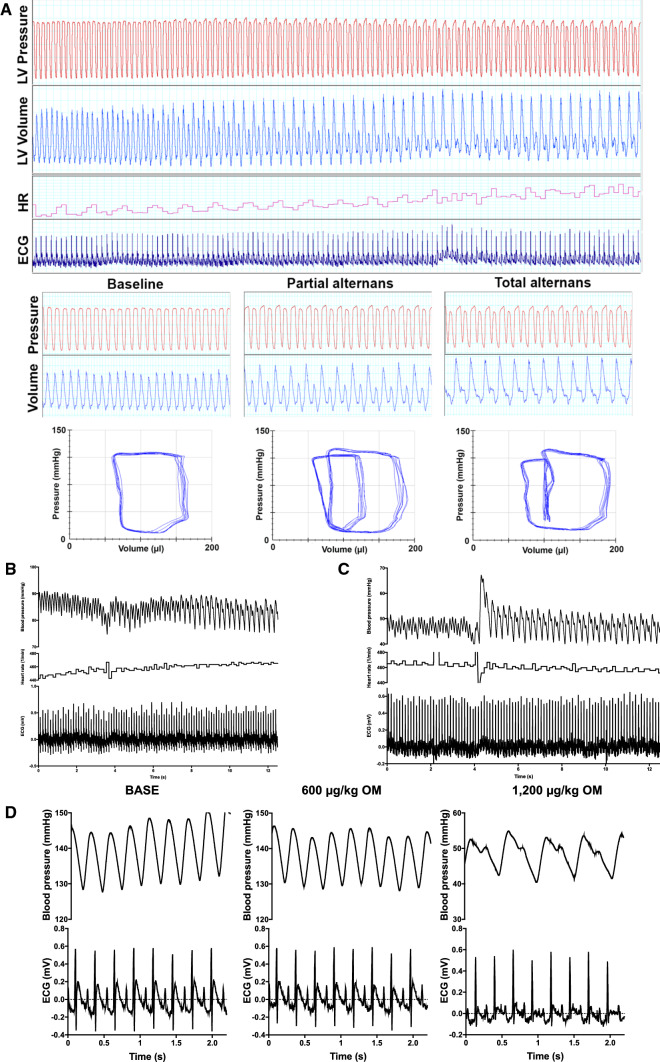
Omecamtiv mecarbil evokes transient *pulsus alternans* in the rat. High doses (1200 µg/kg body weight cumulative dose) of omecamtiv mecarbil (OM) evoked a transient (short-lasting, recurring) electromechanical alternans (alternating pulseless electrical activity) in the rat. It was observed during left ventricular (LV) pressure–volume analysis (**a**) and invasive blood pressure measurements (**b**–**d**). The features and development of the electromechanical alternans are shown by representative experiments. An individual, representative recording of the parallel measurement of LV pressure, volume, heart rate (HR) and the electrocardiogram (ECG) are shown in the upper row of traces in panel (**a**). Features at baseline and on the development of partial and total alternans are shown in the second row of traces in panel (**a**), which illustrate the raw pressure and volume values and the loops. The development of the alternans is also shown in representative recordings by means of invasive arterial blood pressure measurements (**b**, **c**). The electromechanical alternans developed upon a slight increase in heart rate (**a**, **b**) or upon an extrasystole (**c**). Irrespective of its initiation, alternans was characterized by pulseless electrical activity that repeatedly appeared after an normal beat, as represented by the parallel-pressure waveform and ECG recordings at baseline and with a cumulative OM dose of 1200 µg/kg (**d**). Single, representative recordings are shown and were specifically chosen to illustrate this feature

OM evoked transient, periodic, electromechanical alternations. These were characterized by the occurrence of oscillation between normal and low or missing ejections on a beat-to-beat basis (Fig. 5a). This feature was observed in 23 of the 30 rats tested. Alternating contractile dysfunction manifested in either a partial or in a complete form, differentiated by the diastolic filling of the left ventricle. Partial alternans was defined when a normal systole was followed by partial filling and a consequently reduced stroke volume of the LV chamber. Complete alternans was characterized by virtually no filling and a consequent frustrated contraction after the normal systole (Fig. 5a).

In vivo intra-arterial measurement of systemic blood pressure after administration of high-dose OM (1200 µg/kg BW) shed light on the initiation of these transient periods. They started gradually, either in parallel with an increase in heart rate (Fig. 5b) or else were initiated by an extra beat (Fig. 5c). They appeared as alternating increases in arterial blood pressure, similar to a clinical feature known as *pulsus alternans* (Fig. 5d). This phenomenon was not observed with lower OM doses.

**Fig. 6:**
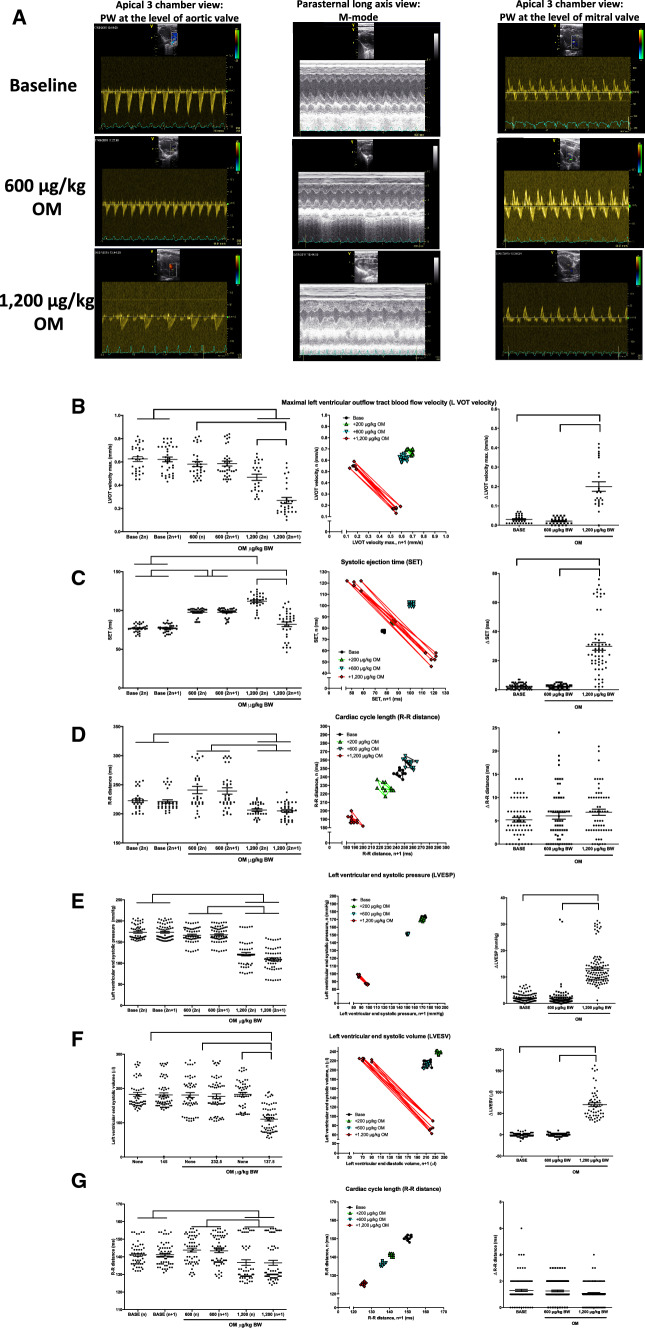
Echocardiographic features of omecamtiv mecarbil evoked transient, periodic electromechanical alternans in the rat. Echocardiography (**a**–**d**) and left ventricular pressure–volume relationships (**e**–**g**) were recorded to evaluate the electromechanical alternans evoked by a 1200 µg/kg body weight (BW) dose of omecamtiv mecarbil (OM). Representative echocardiographic recordings are shown in panel (**a**). Each symbol represents an individual measurement (12 consecutive cardiac cycles in each animal), together with the mean and standard error, in the graphs on the left-hand side. The beat-to-beat variability is shown on the Poincaré plots in the middle set of graphs, where the value for the actual beat is plotted as the function of the value at the next beat in an individual representative recording. On the Poincaré plots, consecutive values are connected by lines. The cumulative dose of OM is indicated on the horizontal axes or in the insets (Poincaré plots). The difference between the consecutive beat pairs is plotted in the graphs on the right-hand side. Significant differences (*P* < 0.05) between the five to six (echocardiography) and nine (pressure–volume analysis) biological replicates are indicated by the braces. Left ventricular outflow tract velocity (**b**) and left ventricular systolic ejection time (**c**) were determined by pulsatile Doppler at the level of the aortic valve in an apical 3 chamber view, while the cardiac cycle length (**d**) was determined by a parallel electrocardiography recording. The characteristics of the electromechanical alternans were also recorded by means of left ventricular pressure–volume relationships, with data plotted similarly to the echocardiography data (**e**–**g**). Left ventricular end-systolic pressure (**e**) and left ventricular end-diastolic volume (**f**) were determined by means of a probe inserted into the left ventricle, whereas the cardiac cycle length (**g**) was determined from the parallel ECG recording

Echocardiography (Fig. 6a–d) and direct LV P–V recordings (Fig. 6e–g) were carried out to characterize this oscillation between normal and reduced contractions. The oscillating nature of the contractions can best be appreciated on Poincaré plots as two distinct groups of data points (representing the normal and the consecutive frustrated contractions) for the parameters derived from echocardiography and P–V recordings. Numerically, the alternations resulted in higher dispersion of the differences between the consecutive parameter values. The maximal LVOT velocity alternated on a beat-to-beat basis from 0.47 ± 0.03 mm/s for the normal beats to 0.27 ± 0.03 mm/s during the consecutive frustrated beats at an OM dose of 1200 µg/kg BW (Fig. 6b) and was reflected by higher dispersion of the differences between the consecutive values (from 0.030 ± 0.004 to 0.200 ± 0.024 mm/s; Fig. 6b). Similarly, SET showed a pronounced alternation at OM 1200 µg/kg BW from 111.9 ± 1.8 ms for the normal beats to 82.1 ± 3.0 ms for the consecutive altered beats (Fig. 6c) and higher dispersion of the differences between the consecutive values (from 2.3 ± 0.2 and 29.8 ± 2.7 ms). In contrast, no alternation in the cardiac cycle length (R-to-R distance) was observed (Fig. 6d).

Functional features of this alternation were further studied by direct P–V recordings (Fig. 6e–g). LV end-systolic pressure values decreased from 172 ± 2 to 92 ± 6 mmHg (Fig. 6e) and a significant alternation in the values was observed (basal beat-to-beat variability increased from 2.0 ± 0.2 to 13.1 ± 0.6 mmHg) at an OM dose of 1200 µg/kg BW. LV end-systolic volume (LVESV) also showed a prominent alternating behavior at an OM dose of 1200 µg/kg BW, as represented by differences in the consecutive beats: 183 ± 6 µL for the normal beats and 111 ± 5 µL for the consecutive altered beats (Fig. 6f). This feature was particularly apparent on the Poincaré plot and was represented by the higher dispersion of the differences among the consecutive values (from − 0.5 ± 0.7 to 71 ± 5 µl). Despite these robust functional alternations, heart rhythm generation was not affected. The R-R distance decreased from 141 ± 1 to 137 ± 1 ms (*P* < 0.05) without an alteration in R-to-R duration: the value of the R–R distance was 137 ± 2 ms after the normal beats and 137 ± 1 ms after the consecutive altered beat at an OM dose of 1200 µg/kg BW (Fig. 6g).

**Fig. 7:**
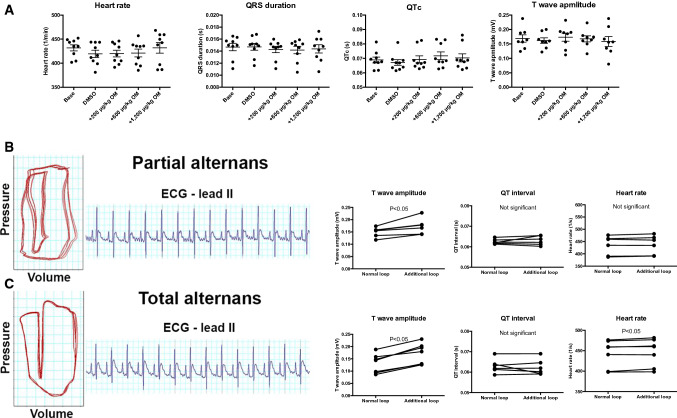
Omecamtiv mecarbil evokes a transient T-wave alternans in the rat. Cardiac electrocardiograms were recorded in rats. A representative tracing and pooled analysis are shown. Heart rate, QRS duration, corrected QT interval (QTc; Bazett formula) and T-wave amplitude were recorded and plotted in periods without electromechanical alternans in the presence of 1200 µg/kg body weight omecamtiv mecarbil (OM) (**a**). Symbols represent individual values from eight to nine replicates. The mean and standard error of the mean are shown in the scatter plots. No statistical differences were found between the groups. Next, cardiac electrocardiography (ECG) parameters were evaluated during the transient periods when partial or total electromechanical alternans was present (**b**). Representative pressure–volume loops and ECG recordings are shown on the left-hand side. The graphs on the right-hand side represent the T-wave amplitudes, QT interval and heart rate values in the normal and consecutive diminished (additional) loops. Symbols represent the individual values determined from six biological replicates. Significant differences are indicated in the graphs

A detailed evaluation of the electrocardiographic recordings revealed no effects of OM 1200 µg/kg BW on heart rate, QRS duration, corrected QT interval or T-wave amplitude in the periods without alternations (Fig. 7a). However, a prominent T-wave alternans was observed in cases where electromechanical alternation was present (Fig. 7b). Moreover, the cardiac cycle seemed to be longer during the apparently effective (normal) contractions, as represented by the lower heart rate when complete electromechanical alternans was present (Fig. 7c).

### *Omecamtiv mecarbil alters intracellular Ca*^*2*+^*handling in canine cardiomyocytes *in vitro

**Fig. 8:**
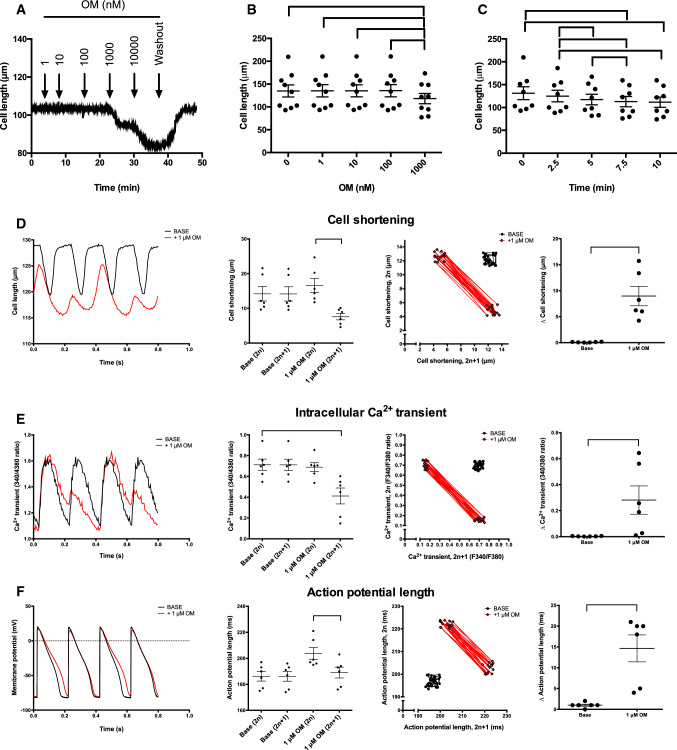
Omecamtiv mecarbil affects Ca^2+^ cycling in isolated intact canine left ventricular cardiomyocytes. Representative recordings are shown in panels (**a**–**c**). The cell lengths were evaluated after 10 min of incubation with the indicated concentrations (0–1000 nM; *n* = 9; **b**) of omecamtiv mecarbil (OM). The kinetics of 1 μM OM evoked unloaded (occurring without stimulation) reduction of diastolic length of cardiomyocytes, as shown in panel (**c**). Each symbol represents a measurement on an individual cell. The mean is represented by horizontal lines and the error bars represent the standard error of the mean. In the second set of experiments, the cell length (optical measurement; **d**), intracellular Ca^2+^ concentration (measurement of changes in FURA-2 fluorescence intensity ratios; **e**) and membrane potential (measured by patch clamp; **f**) were measured in parallel on the same isolated cardiomyocyte. The stimulation frequency was 5 Hz. The recordings before OM treatment are labeled ‘BASE’, while the traces recorded from the same cell in the presence of OM are labeled ‘ + 1 μM OM’. Representative recordings are shown in the graphs on the left-hand side. Six biological replicates were recorded for all parameters and evaluated. Each symbol represents a single measurement on an individual cell. The mean is represented by horizontal lines and the error bars represent the standard error of the mean. To visualize the variations in consecutive values, Poincaré plots were constructed involving 30 consecutive cycles. In these plots, the parameter values of the even-numbered cycles (2n) are plotted as a function of the value of the next odd-numbered cycle (2n + 1). Finally, in the right-hand graphs, each symbol represents the average variability of an individual cell. Significant differences (*P* < 0.05) between groups are shown by the braces

The electrophysiological effects of OM were studied at the cellular level using isolated canine LV cardiomyocytes, as they provide the best model for humans (Fig. 8). Treatment with 1 µM OM increased the stiffness of unloaded cells (representative example shown in Fig. 8a). Unstimulated cardiomyocytes shrank from 131 ± 14 to 112 ± 11 µm (Fig. 8b) in the presence of 1 µM OM. This OM-evoked reduced diastolic length developed within 10 min of application (Fig. 8c). Stimulation of isolated LV cardiomyocytes (faster than 3 Hz) resulted in the occurrence of oscillation between normal and reduced contractions on a beat-to-beat basis, similar to the in vivo measurements. Alternation was considered to be present when a difference of ≥ 10% occurred between the consecutively recorded stimulated cell shortenings. This alternation was present in three out of 14 cells at 4 Hz and in nine out of 14 at 5 Hz. The alternation was studied at 5 Hz by recording cell length and changes in intracellular Ca^2+^ concentrations and membrane potential (AP) in parallel (Fig. 8d–f). There was no difference in contraction (decrease in cell length) before OM treatment in the consecutive contractions (14.20 ± 2.09 and 14.16 ± 2.06 µm), while a significant difference developed upon OM administration (16.6 ± 2.0 and 7.6 ± 0.9 µm; Fig. 8d). This resulted in prominent signs of alternation on the Poincaré plot and was represented by higher dispersion of the differences among the consecutive values (from 0.09 ± 0.03 to 8.97 ± 1.9 µm in the presence of 1 µM OM; Fig. 8d). In parallel, the amplitude of Ca^2+^ transients (CaT amplitude) decreased at the diminished contractions from 0.69 ± 0.04 to 0.41 ± 0.08 (Fig. 8e) in the presence of 1 µM OM, which was again apparent on the Poincaré plot and as was represented by higher dispersion of the differences among the consecutive values (from 0.002 ± 0.0007 to 0.28 ± 0.11; Fig. 8e). In line with this, long APs (representing normal contractions) alternated with unaffected APs (representing the diminished contractions) (action potential duration at 90% repolarization, APD_90_ alternated between 204 ± 4 and 189 ± 4 ms; Fig. 8f), a feature which was particularly apparent on the Poincaré plot and as marked dispersion (Fig. 8f) There was no difference in restoration of the APD upon OM treatment, suggesting no involvement of ionic channel recovery (see the supplementary material).

## Discussion

In this study, we have reported two previously unrecognized features of OM that overlap with its positive inotropic effect. The first appears to be an on-target side effect that severely impairs the diastolic filling of the heart. The second is a periodic electromechanical alternation (present at higher OM doses) in which normal beats alternate with diminished cardiac contractions on a beat-to-beat basis, a feature similar to *pulsus alternans*.

In recent clinical trials, OM was tested by pharmacokinetic titrations, resulting in a plasma concentration of 318 ± 129 ng/mL (representing 0.79 µM) [27]. In the most recent clinical method for pharmacokinetic adjustment, the daily dose of OM administered was increased in patients with an OM plasma concentration < 300 ng/mL (0.75 µM), but decreased above 1000 ng/mL (2.49 µM) [26]. Note that doses > 1200 ng/mL (about three times higher than those in our in vitro experiments) were previously reported to lead to excessive prolongation of the systole, thus limiting coronary blood flow during diastole, and possibly leading to myocardial ischemia [4]. The maximum intravenously administered dose of OM used in our experiments accorded with clinical applications (initial dose of 1000 µg/kg BW within the first hour) [3].

In our in vitro experiments, cellular effects were studied at an OM concentration of 401 ng/mL (1 µM), which overlaps with the reported effective clinical serum concentrations of the drug [3, 5, 25–28] We also confirmed our previous finding [1] of robust Ca^2+^ sensitization when OM was administered at the therapeutic concentration. This effect was paralleled by slower kinetics for both contraction and relaxation, and by elevated passive stiffness.

Regarding our in vivo results, the positive effects of OM on the systolic function of the rat (as indicated by ejection time, EF, ESPVRq and PRSW) occurred at a dosage of 600 µg/kg BW. Interestingly, some diastolic parameters (E:A, IVRT and τ_w_) were affected by the lowest dose of OM (200 µg/kg). It is important to note that improvement in EF (one of the major systolic parameters considered in human heart failure) was observed without a marked increase in stroke volume. This raises the question of whether improvement in EF alone should be considered as a favorable outcome in heart failure studies with OM. Here, we found that the major contributor to improved EF was the reduced diastolic volume of the LV chamber, which did not result in a marked improvement in LV systolic function but was paralleled by prominent diastolic dysfunction.

Cellular contractility, Ca^2+^ transient and AP analysis were performed in isolated canine LV cardiomyocytes. These were chosen because their electrophysiological properties closely resemble those of humans [22, 23]. OM treatment mimicked positive inotropy (improved shortening) and diastolic dysfunction (reduced diastolic length) in isolated canine cardiomyocytes, validating these cells for further experiments. The potential mechanisms of these alterations were studied in this cellular model to test the potential involvement of Ca^2+^ handling and electrophysiological properties. OM reduced the length of these unloaded cardiomyocytes in unstimulated (diastolic) conditions. This is compatible with the finding that OM increased the passive stiffness of permeabilized human cardiac myocytes in an apparently Ca^2+^-independent mechanism, as well as slowing down relaxation after contraction. Our data suggest that the level of increased Ca^2+^ sensitivity may be extreme: both reduced diastolic length and increased cardiomyocyte passive stiffness occurred at very low (diastolic) Ca^2+^ concentrations.

It was proposed in previous reports [12, 13] that OM may lead to diastolic dysfunction, a possibility addressed by the developers of the drug using data collected in the Chronic Oral Study of Myosin Activation to Increase Contractility in Heart Failure (COSMIC-HF). That post hoc analysis showed an increase in IVRT without changes in E:A ratio or E wave (abstract RF299 presented at the American Heart Association Scientific Sessions, 16–18 November 2019, Philadelphia, PA). Values for left atrial diameter, or the potential occurrence of *pulsus alternans* and T-wave alternans were not mentioned in the clinical studies.

The increase in SET can be explained by the apparent increase in the Ca^2+^ sensitivity of force production: i.e. low cytoplasmic Ca^2+^ concentrations are sufficient for both the initiation and maintenance of contraction. However, we also identified a marked reduction in the rate of force generation and relaxation under conditions where the Ca^2+^ concentration reached a steady state in permeabilized human cardiomyocytes, suggesting a previously unidentified action of OM on the Ca^2+^ regulation of the actin–myosin complex. It appears that both Ca^2+^-dependent activation and dissociation are much slower in the presence of OM. OM also increased the resting stiffness of cardiomyocytes. This can be explained by (1) a Ca^2+^-independent initiation of force production or (2) a direct interaction of OM with the contractile protein machinery, in addition to the myosin motor. These cellular data suggest an alternative mechanism for the clinically observed decrease in LV diameter: it may be the result of Ca^2+^ sensitization of contraction occurring at diastolic Ca^2+^ concentrations, similar to that evoked by the Ca^2+^-sensitizer EMD 57,033 [9]. Additionally, we confirmed that OM does not affect intracellular Ca^2+^ transients at low concentrations. In contrast, OM affected the APD and T-wave morphology at higher concentrations. These latter, previously unidentified features can be explained by (1) altered ryanodine receptor (RyR) function and (2) slower dissociation of Ca^2+^ from the troponin complex.

The second major novelty of our work is that OM evoked a dose- and apparently heart rate-dependent, transient, electromechanical alternation, a feature similar to *pulsus alternans*, in the rat. This was present in 23 of 30 rats at a high OM dose (1200 µg/kg). It occurred during periods of slightly elevated heart rates or higher stimulation frequencies in isolated canine LV cardiac myocytes (nine of 14 cells at a stimulation frequency of 5 Hz), suggesting that this feature is heart rate-dependent but seems to be independent of species. This alternating cardiac performance involved a mechanically normal systole with prolonged ejection, such that the left ventricle was unable to relax sufficiently to be filled before the following systole. This resulted in an incomplete systole, without major changes in QRS morphology or corrected QT interval, but with an alternating pattern of T waves. T-wave alternans has been described in heart failure patients, mostly during progression of the disease. It has been suggested that T-wave alternans can be responsible for the initiation of life-threatening arrhythmias [20]. Diaz et al. suggested that alteration of the RyR opening probability is responsible for T-wave alternans [4], a view aligned with the results of Nanasi et al. who described that OM changes the opening probability of RyR [15].

In addition to T-wave alternans, the Ca^2+^ sensitization evoked by OM may also lead to an increased probability of ventricular arrhythmias. Baudenbacher et al. [2] showed that certain mutations in troponin T (TnT) and troponin I genes might create a myocardial substrate for ventricular arrhythmias through Ca^2+^ sensitization. Arrhythmias were evoked by the Ca^2+^ sensitizer EMD 57,033 and prevented by blebbistatin (a myofilament Ca^2+^ desensitizer). The same authors also proposed that Ca^2+^ sensitization provokes ventricular arrhythmias through Ca^2+^ transient alternans, which is exactly what we found with OM. Moreover, it was also shown by TnT mutations that alterations in Ca^2+^ sensitization may lead to changes in AP characteristics [29]. Indeed, we found changes in AP characteristics upon OM treatment, such as an alternating pattern of AP length. Moreover, several groups of researchers have discussed the role of Ca^2+^ alternans leading to increased dispersion of excitability and refractoriness of myocytes, which may serve as a substrate for re-entry mechanisms and thus ventricular arrhythmias [8, 19, 30]. Our in vivo experiments in the rat also showed a dose-dependent severe decrease in blood pressure, which might be the result of severe diastolic dysfunction and/or the alternans.

Use of oral OM treatment in patients with heart failure with reduced LV ejection fraction in the GALACTIC-HF study [26], showed only a moderate clinical benefit despite its well-documented positive effects on systolic function [3, 25, 27]. Our present data, which imply that improvements in systolic function can be offset or attenuated by OM-evoked negative effects on diastolic function, may outline an explanation for those clinical findings. Unfortunately, the GALACTIC-HF trial data do not allow a detailed assessment of diastolic function [26]. Further clinical investigations are therefore required to address hypothetical alterations in diastolic function upon administration of OM. In particular, we propose that echocardiographic indices of IVRT, left atrial size and Tei index should be considered for future patient selection and personalized OM treatments. It is important to emphasize that no signs of electromechanical alternations or increases in the number of life-threatening arrhythmias have been reported when rigorous and regular plasma concentration measurements of OM have been applied in clinical practice. Nevertheless, OM appears to be effective only in patients without atrial fibrillation or atrial flutter, suggesting an interaction between cardiac rhythmicity and the clinical efficacy of OM treatments [26].

When considering the limitations of our study, we must highlight the fact that the in vivo experiments were performed in rats, while the in vitro ones were performed in isolated canine and human cardiac myocytes. Caution is appropriate when extrapolating experimental data to possible human outcomes. It needs also to be noted that all the experimental data were obtained in healthy animals. It is known, however, that canine cardiac myocytes are good models of human electrophysiology. Finally, the results showed a severe drop in blood pressure values in response to OM treatment without increases in heart rate, which raises the possibility of an effect of anesthesia on the in vivo cardiovascular parameters (i.e. a blunted baroreflex may dispose the rats to develop hypotension).

## Supplementary Information

Below is the link to the electronic supplementary material.

Supplementary file1 (PPTX 68 KB)

Supplementary file2 (DOCX 40 KB)

## References

[1] Bakkehaug JP, Kildal AB, Engstad ET, Boardman N, Naesheim T, Ronning L, Aasum E, Larsen TS, Myrmel T, How OJ (2015). Myosin activator omecamtiv mecarbil increases myocardial oxygen consumption and impairs cardiac efficiency mediated by resting myosin ATPase activity. Circ Heart Fail.

[2] Baudenbacher F, Schober T, Pinto JR, Sidorov VY, Hilliard F, Solaro RJ, Potter JD, Knollmann BC (2008). Myofilament Ca2+ sensitization causes susceptibility to cardiac arrhythmia in mice. J Clin Invest.

[3] Cleland JG, Teerlink JR, Senior R, Nifontov EM, Mc Murray JJ, Lang CC, Tsyrlin VA, Greenberg BH, Mayet J, Francis DP, Shaburishvili T, Monaghan M, Saltzberg M, Neyses L, Wasserman SM, Lee JH, Saikali KG, Clarke CP, Goldman JH, Wolff AA, Malik FI (2011). The effects of the cardiac myosin activator, omecamtiv mecarbil, on cardiac function in systolic heart failure: a double-blind, placebo-controlled, crossover, dose-ranging phase 2 trial. Lancet.

[4] Diaz ME, Eisner DA, O'Neill SC (2002). Depressed ryanodine receptor activity increases variability and duration of the systolic Ca2+ transient in rat ventricular myocytes. Circ Res.

[5] Greenberg BH, Chou W, Saikali KG, Escandon R, Lee JH, Chen MM, Treshkur T, Megreladze I, Wasserman SM, Eisenberg P, Malik FI, Wolff AA, Shaburishvili T (2015). Safety and tolerability of omecamtiv mecarbil during exercise in patients with ischemic cardiomyopathy and angina. JACC Heart Fail.

[6] Horvath B, Szentandrassy N, Veress R, Almassy J, Magyar J, Banyasz T, Toth A, Papp Z, Nanasi PP (2017). Frequency-dependent effects of omecamtiv mecarbil on cell shortening of isolated canine ventricular cardiomyocytes. Naunyn Schmiedebergs Arch Pharmacol.

[7] Kovacs A, Fulop GA, Kovacs A, Csipo T, Bodi B, Priksz D, Juhasz B, Beke L, Hendrik Z, Mehes G, Granzier HL, Edes I, Fagyas M, Papp Z, Barta J, Toth A (2016). Renin overexpression leads to increased titin-based stiffness contributing to diastolic dysfunction in hypertensive mRen2 rats. Am J Physiol Heart Circ Physiol.

[8] Landstrom AP, Dobrev D, Wehrens XHT (2017). Calcium signaling and cardiac arrhythmias. Circ Res.

[9] Lee JA, Allen DG (1997). Calcium sensitisers: mechanisms of action and potential usefulness as inotropes. Cardiovasc Res.

[10] Magyar J, Szentandrassy N, Banyasz T, Kecskemeti V, Nanasi PP (2004). Effects of norfluoxetine on the action potential and transmembrane ion currents in canine ventricular cardiomyocytes. Naunyn Schmiedebergs Arch Pharmacol.

[11] Malik FI, Hartman JJ, Elias KA, Morgan BP, Rodriguez H, Brejc K, Anderson RL, Sueoka SH, Lee KH, Finer JT, Sakowicz R, Baliga R, Cox DR, Garard M, Godinez G, Kawas R, Kraynack E, Lenzi D, Lu PP, Muci A, Niu C, Qian X, Pierce DW, Pokrovskii M, Suehiro I, Sylvester S, Tochimoto T, Valdez C, Wang W, Katori T, Kass DA, Shen YT, Vatner SF, Morgans DJ (2011). Cardiac myosin activation: a potential therapeutic approach for systolic heart failure. Science.

[12] Mamidi R, Li J, Gresham KS, Verma S, Doh CY, Li A, Lal S, Dos Remedios CG, Stelzer JE (2017). Dose-dependent effects of the myosin activator omecamtiv mecarbil on cross-bridge behavior and force generation in failing human myocardium. Circ Heart Fail.

[13] Molnar A, Borbely A, Czuriga D, Ivetta SM, Szilagyi S, Hertelendi Z, Pasztor ET, Balogh A, Galajda Z, Szerafin T, Jaquet K, Papp Z, Edes I, Toth A (2009). Protein kinase C contributes to the maintenance of contractile force in human ventricular cardiomyocytes. J Biol Chem.

[14] Nagy L, Kovacs A, Bodi B, Pasztor ET, Fulop GA, Toth A, Edes I, Papp Z (2015). The novel cardiac myosin activator omecamtiv mecarbil increases the calcium sensitivity of force production in isolated cardiomyocytes and skeletal muscle fibres of the rat. Br J Pharmacol.

[15] Nanasi P, Gaburjakova M, Gaburjakova J, Almassy J (2017). Omecamtiv mecarbil activates ryanodine receptors from canine cardiac but not skeletal muscle. Eur J Pharmacol.

[16] Planelles-Herrero VJ, Hartman JJ, Robert-Paganin J, Malik FI, Houdusse A (2017). Mechanistic and structural basis for activation of cardiac myosin force production by omecamtiv mecarbil. Nat Commun.

[17] Radovits T, Olah A, Lux A, Nemeth BT, Hidi L, Birtalan E, Kellermayer D, Matyas C, Szabo G, Merkely B (2013). Rat model of exercise-induced cardiac hypertrophy: hemodynamic characterization using left ventricular pressure-volume analysis. Am J Physiol Heart Circ Physiol.

[18] Rohde JA, Thomas DD, Muretta JM (2017). Heart failure drug changes the mechanoenzymology of the cardiac myosin powerstroke. Proc Natl Acad Sci U S A.

[19] Sato D, Shiferaw Y, Garfinkel A, Weiss JN, Qu Z, Karma A (2006). Spatially discordant alternans in cardiac tissue: role of calcium cycling. Circ Res.

[20] Sipido KR (2004). Understanding cardiac alternans: the answer lies in the Ca2+ store. Circ Res.

[21] Swenson AM, Tang W, Blair CA, Fetrow CM, Unrath WC, Previs MJ, Campbell KS, Yengo CM (2017). Omecamtiv mecarbil enhances the duty ratio of human beta-cardiac myosin resulting in increased calcium sensitivity and slowed force development in cardiac muscle. J Biol Chem.

[22] Szabo G, Szentandrassy N, Biro T, Toth BI, Czifra G, Magyar J, Banyasz T, Varro A, Kovacs L, Nanasi PP (2005). Asymmetrical distribution of ion channels in canine and human left-ventricular wall: epicardium versus midmyocardium. Pflugers Arch.

[23] Szentadrassy N, Banyasz T, Biro T, Szabo G, Toth BI, Magyar J, Lazar J, Varro A, Kovacs L, Nanasi PP (2005). Apico-basal inhomogeneity in distribution of ion channels in canine and human ventricular myocardium. Cardiovasc Res.

[24] Szentandrassy N, Horvath B, Vaczi K, Kistamas K, Masuda L, Magyar J, Banyasz T, Papp Z, Nanasi PP (2016). Dose-dependent electrophysiological effects of the myosin activator omecamtiv mecarbil in canine ventricular cardiomyocytes. J Physiol Pharmacol.

[25] Teerlink JR, Clarke CP, Saikali KG, Lee JH, Chen MM, Escandon RD, Elliott L, Bee R, Habibzadeh MR, Goldman JH, Schiller NB, Malik FI, Wolff AA (2011). Dose-dependent augmentation of cardiac systolic function with the selective cardiac myosin activator, omecamtiv mecarbil: a first-in-man study. Lancet.

[26] Teerlink JR, Diaz R, Felker GM, McMurray JJV, Metra M, Solomon SD, Adams KF, Anand I, Arias-Mendoza A, Biering-Sorensen T, Bohm M, Bonderman D, Cleland JGF, Corbalan R, Crespo-Leiro MG, Dahlstrom U, Echeverria LE, Fang JC, Filippatos G, Fonseca C, Goncalvesova E, Goudev AR, Howlett JG, Lanfear DE, Li J, Lund M, Macdonald P, Mareev V, Momomura SI, O'Meara E, Parkhomenko A, Ponikowski P, Ramires FJA, Serpytis P, Sliwa K, Spinar J, Suter TM, Tomcsanyi J, Vandekerckhove H, Vinereanu D, Voors AA, Yilmaz MB, Zannad F, Sharpsten L, Legg JC, Varin C, Honarpour N, Abbasi SA, Malik FI, Kurtz CE, Investigators G-H (2021). Cardiac myosin activation with omecamtiv mecarbil in systolic heart failure. N Engl J Med.

[27] Teerlink JR, Felker GM, McMurray JJ, Solomon SD, Adams KF, Cleland JG, Ezekowitz JA, Goudev A, Macdonald P, Metra M, Mitrovic V, Ponikowski P, Serpytis P, Spinar J, Tomcsanyi J, Vandekerckhove HJ, Voors AA, Monsalvo ML, Johnston J, Malik FI, Honarpour N, Investigators C-H (2016). Chronic oral study of myosin activation to increase contractility in heart failure (COSMIC-HF): a phase 2, pharmacokinetic, randomised, placebo-controlled trial. Lancet.

[28] Teerlink JR, Felker GM, McMurray JJV, Ponikowski P, Metra M, Filippatos GS, Ezekowitz JA, Dickstein K, Cleland JGF, Kim JB, Lei L, Knusel B, Wolff AA, Malik FI, Wasserman SM, Investigators A-A (2016). Acute treatment with omecamtiv mecarbil to increase contractility in acute heart failure: the ATOMIC-AHF study. J Am Coll Cardiol.

[29] Wang L, Kryshtal DO, Kim K, Parikh S, Cadar AG, Bersell KR, He H, Pinto JR, Knollmann BC (2017). Myofilament calcium-buffering dependent action potential triangulation in human-induced pluripotent stem cell model of hypertrophic cardiomyopathy. J Am Coll Cardiol.

[30] Weiss JN, Garfinkel A, Karagueuzian HS, Nguyen TP, Olcese R, Chen PS, Qu Z (2015). Perspective: a dynamics-based classification of ventricular arrhythmias. J Mol Cell Cardiol.

[31] Woody MS, Greenberg MJ, Barua B, Winkelmann DA, Goldman YE, Ostap EM (2018). Positive cardiac inotrope omecamtiv mecarbil activates muscle despite suppressing the myosin working stroke. Nat Commun.

