# Supplementary figures and images for: Omecamtiv mecarbil evokes diastolic dysfunction and leads to periodic electromechanical alternans
Source: Basic Res Cardiol. 2021 Apr 12;116(1):24. doi: 10.1007/s00395-021-00866-8 (PMC8041714; doi:10.1007/s00395-021-00866-8)

## Slide 1
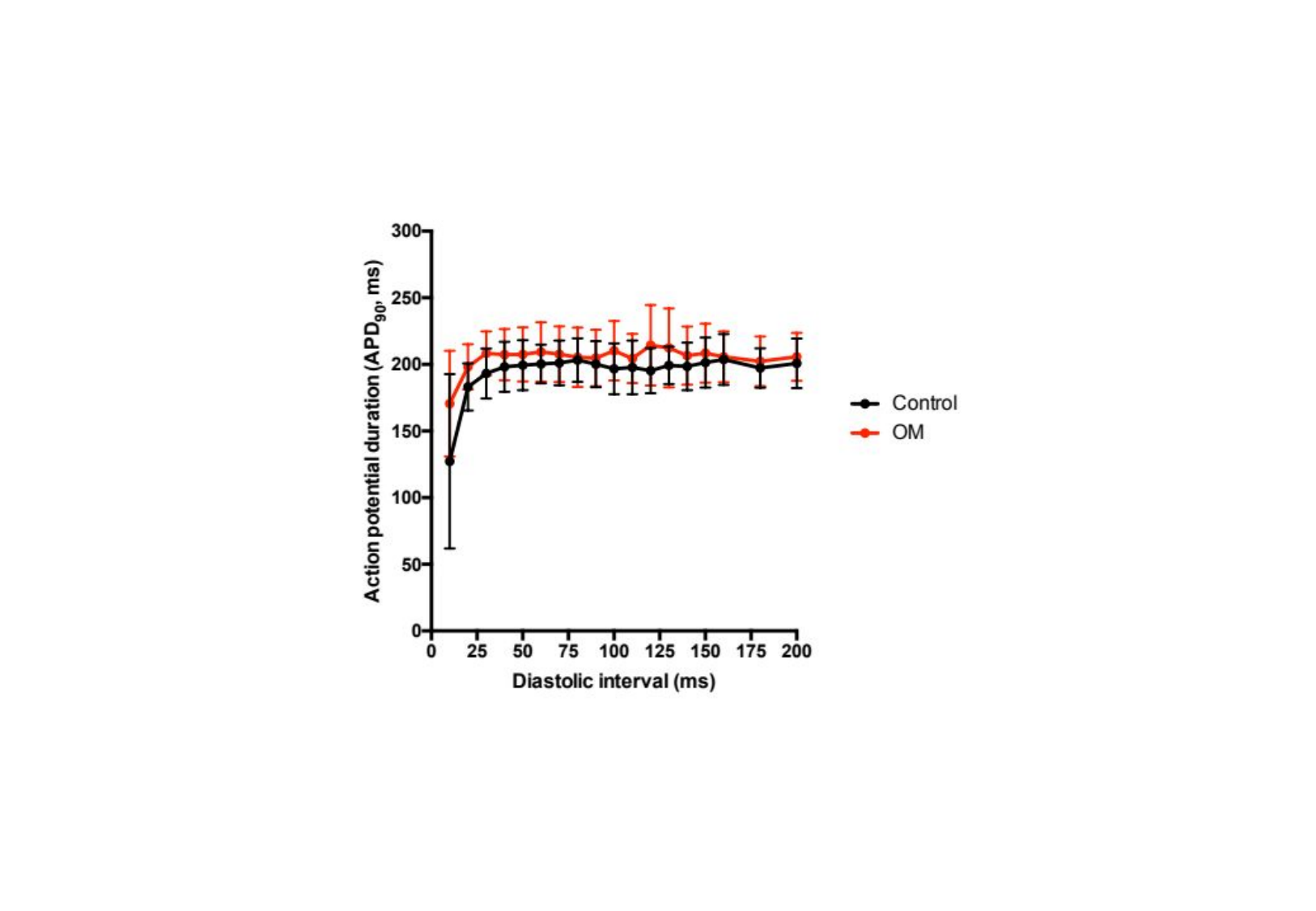

Supplement: Supplementary file 1 — Supplementary file1 (PPTX 68 KB) [file 395_2021_866_MOESM1_ESM.pptx]
