# Supplementary material for: Omecamtiv mecarbil evokes diastolic dysfunction and leads to periodic electromechanical alternans
Source: Basic Res Cardiol. 2021 Apr 12;116(1):24. doi: 10.1007/s00395-021-00866-8 (PMC8041714; doi:10.1007/s00395-021-00866-8)
Supplement: Supplementary file 2 — Supplementary file2 (DOCX 40 KB) [file 395_2021_866_MOESM2_ESM.docx]

Full description of the applied Methods

*Animal experiments:*

All animal care and experimental procedures were approved by the Ethical Committee of the University of Debrecen (Ethical Statement No. 1/2013/DE MAB) and conformed to Directive 2010/63/EU of the European Parliament. Investigations were carried out on male 13-15 week-old Wistar-Kyoto rats, weighing 300-330 g (Toxi-Coop Toxicological Research Centre, Dunakeszi, Hungary). Myocytes were isolated from Mongrel dogs (described below). The animals were kept at constant temperature rooms (23±0.2°C) with a 12-h/12-h light/dark cycle and were fed a standard chow and drank tap water ad libitum. All procedures were as humane as possible to minimize the distress of the animals.

*Hemodynamic measurements:*

In vivo hemodynamic measurements were performed as described previously [6]. Briefly, rats were anesthetized with pentobarbital sodium (60 mg/kg BW), tracheotomized, and intubated to facilitate breathing. Animals were placed on controlled heating pads, and the core temperature, measured via a rectal probe, was maintained at 37°C. A polyethylene catheter was inserted into the left external jugular vein for fluid and OM administration. A 2-Fr pressure-conductance microcatheter (SPR-838; Millar Instruments, Houston, TX, USA) was inserted into the right carotid artery and advanced into the ascending aorta.

The catheter was thereafter advanced into the LV under pressure control. Steady-state P-V relations were recorded, after stabilization for 10 min, Signals were continuously registered at a sampling rate of 1,000 samples/s using a P-V conductance system (MPVS-Ultra; Millar Instruments) connected to the PowerLab 16/30 data acquisition system (AD Instruments, Colorado Springs, CO, USA), stored and displayed on a personal computer by the LabChart7 Software System (AD Instruments). A special P-V analysis tool (PVAN, Millar Instruments) was used to compute and calculate hemodynamic parameters: LV end-systolic pressure (ESP), LV end-diastolic pressure (EDP), LV end-diastolic volume (EDV), LV end-systolic volume (ESV) and diastolic pressure decrement (dP/dt_min_), time constant of LV pressure decay (Tau_W_; according to the Weiss method), ejection fraction (EF), stroke volume (SV).

LV P-V relations were determined at different preloads during transient occlusion of the inferior caval vein to obtain load-independent parameters of LV systolic and diastolic function. The slope of end-systolic P-V relationship (ESPVR_q_, according to the curvilinear model) and preload recruitable stroke work (PRSW) were calculated as load-independent indices of LV contractility. The slope of the LV end-diastolic P-V relationship (EDPVR) was calculated as a reliable indicator of LV stiffness.

After recording the baseline parameters, DMSO (solvent), and three consecutive doses of OM (200, 400 and 600 µg/kg BW) were injected as intravenous bolus, yielding a maximum of 1,200 µg/kg BW cumulative dose. The time between consecutive injections was 12-15 min per dose.

The above described measurements and data analysis were performed at baseline, and 10 minutes after administering DMSO or any dose of OM.

The volume calibration was performed as previously described using saline administration and cuvette calibration [6].

*Blood pressure measurement*

Effects of increasing doses of OM on blood pressure were tested invasively. Effects of DMSO was tested, followed by OM, administered through the cannulated left jugular vein in increasing doses (200, 400 and 600 µg/kg BW), yielding a maximum of 1200 µg/kg BW cumulative dose. Blood pressure was recorded invasively through the cannulation of the right carotid artery (Haemosys, Experimetria, Hungary). ECG signals were recorded simultaneously.

*Echocardiography*

In vivo effects of increasing doses of OM were tested by echocardiography using a General Electric Vivid E9 ultrasound system equipped with a linear 14.1-MHz i13L probe (General Electric, Fairfield, CT) as described earlier [2]. After achieving light anaesthesia with ketamine and xylazine (50 mg/kg and 5 mg/kg, respectively) the rats were placed on a heating pad, where the temperature was maintained with the aid of a rectal probe. The left external jugular vein was cannulated, in order to administer the increasing doses of OM. First, the effects of the solvent, DMSO was tested, and then the increasing doses of OM were injected (200, 400 and 600 µg/kg BW) as a bolus, yielding a maximum of 1,200 µg/kg BW cumulative dose. A complete ultrasound assessment was made after each dose, by a single observer. Ejection fraction (EF), fractional shortening (FS), left ventricular internal diameter in systole (LVIDS) and left ventricular internal diameter in diastole (LVIDD) was assessed by recording a parasternal long axis (PLAX) M-mode at the level of the papillary muscles. Further testing of diastolic function was achieved by Doppler measurement of transmitral flow in apical 3 chamber view at the level of mitral valve, to measure E/A ratio, and isovolumetric relaxation time (IVRT). Early and late annular velocity (e’=mm/s and a’=mm/s respectively) were assessed by tissue Doppler at the level of the mitral annulus. LVOTmax, velocity time integral and systolic ejection time (SET) was measured on images recorded by PW Doppler in the level of the left ventricular outflow tract, just above the aortic valve in apical 3 chamber view. End diastolic left atrial internal area was measured by using a 2D apical 3 chamber view recording. All the echo images were analysed by the EchoPAC software (General Electric) by a single observer.

*Mechanical measurements on permeabilized myocytes*

Isometric force generation of single LV cardiomyocytes was measured at sarcomere lengths of 2.3 μm, according to formerly described method [4]. Briefly, deep frozen (-80°C) human left ventricular the free wall samples (from unimplanted donor hearts) were mechanically disrupted in isolating solution (ISO, containing in mM: KCl 100, [ethylene glycol tetraacetic acid](https://www.sciencedirect.com/topics/medicine-and-dentistry/egtazic-acid) 2, MgCl_2_ 1, Na_2_ATP 4, [imidazole](https://www.sciencedirect.com/topics/medicine-and-dentistry/imidazole) 10; [pH](https://www.sciencedirect.com/topics/biochemistry-genetics-and-molecular-biology/ph) 7.0), then incubated in ISO containing 0.5% Triton X-100 detergent for 5 min, and subsequently washed in ISO again, at 4°C. Myocyte-sized preparations were mounted with silicone adhesive between a high-speed length controller (Aurora Scientific Inc., Aurora, Canada) and a force transducer (SensoNor AS, Horten, Norway) in ISO at 15°C. Cardiomyocyte Ca^2+^-activated force generation was evoked by transferring the preparation from relaxing solution (composition and concentrations in mM: BES, 10.0; KCl, 37.11; MgCl_2_, 6.41; EGTA, 7.0; ATP, 6.94; creatine phosphate, 15.0 at pH 7.2) to activating solution (containing Ca^2+^-EGTA instead of EGTA, otherwise same composition as the relaxing solution). Ca^2+^ concentrations were expressed as –lg[Ca^2+^] units. The pCa of relaxing solution was 9.0 (1 nM free Ca^2+^), whereas the pCa of maximal activating solution was 4.75 (18 μM free Ca^2+^). All solutions were supplemented with protease inhibitors: 500 μM phenylmethylsulfonyl fluoride, 40 μM leupeptin and 10 μM E-64:. All chemicals were purchased from Sigma-Aldrich (St. Louis, MO, USA).

Maximal Ca^2+^-activated force (*F*_max_) was determined at pCa 4.75. Experiments were recorded and analyzed by custom-built LABVIEW Data Acquisition platform and LabVIEW analyzing software (National Instruments Corp., Austin, TX, USA). *F*_active_ values at pCa < 4.75 were normalized to *F*_max_ and fitted to a modified Hill equation in Origin 6.0 analysis program (OriginLab, Northampton, MA, USA) providing the Ca^2+^ sensitivity curve for each individual cell. Accordingly, pCa value for the half-maximal Ca^2+^-induced contraction (pCa_50_) defines the Ca^2+^ sensitivity of force production of the contractile machinery. Following each Ca^2+^-dependent tension development, cardiomyocyte Ca^2+^-independent passive force (*F*_passive_) corresponding to cellular relaxation was measured by the shortening to 80% of initial preparation length for 8 sec in relaxing solution. For every single cell, original forces were normalized to myocyte cross-sectional area indicating absolute force values of *F*_max_, *F*_active_ and *F*_passive_ expressed in kN/m^2^. LV samples from 4 different hearts (5-6 cardiomyocytes from each heart) were used in the force measurements.

*Isolation of single canine ventricular cardiomyocytes for action potential, cell length and Ca^2+^-transient measurements*

Adult mongrel dogs of either sex were anaesthetized with intramuscular injections of 10 mg/kg ketamine hydrochloride (Calypsol, Richter Gedeon, Hungary) and 1 mg/kg xylazine hydrochloride (Sedaxylan, Eurovet Animal Health BV, The Netherlands). The hearts were quickly removed and placed in Tyrode solution containing (in mM) NaCl, 144; KCl, 5; CaCl_2_, 2.5; MgCl_2_, 1.2; HEPES, 5; and dextrose, 11 (pH=7.4, osmolality=295-303 mmol/kg). Single cardiomyocytes were obtained by enzymatic dispersion using the segment perfusion technique, as described previously [3]. Briefly, a wedge-shaped section of the left ventricular wall supplied by the left anterior descending coronary artery was dissected, cannulated and perfused with Tyrode solution. After the removal of blood from the tissue the perfusion was switched to a nominally Ca^2+^-free Joklik solution (Minimum Essential Medium Eagle #M0518; Sigma-Aldrich, St. Louis, MO, USA) for 5 min. This was followed by 30-35 min perfusion with Joklik solution supplemented with 1 mg/ml collagenase (Type II, Worthington, Lakewood, NJ, USA) and 0.2 % bovine serum albumin (Fraction V., Sigma-Aldrich, St. Louis, MO, USA) containing 50 µM Ca^2+^. The left ventricular wall was cut into small pieces and the cell suspension was washed with Joklik solution. After gradually restoring the normal external Ca^2+^ concentration, the cells were stored in Minimum Essential Medium Eagle (product #M0643, Sigma-Aldrich, St. Louis, MO, USA) at 15 ºC until use.

*Recording of action potentials of isolated canine left ventricular cardiomyocytes*

All experiments were performed as previously described [1]. Rod-shaped viable ventricular cardiomyocytes, showing clear striation, were placed in a 1 ml volume experimental chamber mounted on the stage of an inverted microscope (Nikon Diaphot 300; Nikon, Tokyo, Japan). After sedimentation, cardiomyocytes were continuously superfused with at 37 °C Tyrode solution at a rate of 1-2 ml/min. Cells were impaled with 3 M KCl filled conventional borosilicate microelectrodes having tip resistances between 20-40 MΩ, connected to the input of a Multiclamp 700A amplifier (Molecular Devices, Sunnyvale, CA, USA). Action potentials (AP) were elicited through these intracellular electrodes by applying 2 ms wide rectangular current pulses having amplitudes of twice the diastolic threshold. The membrane potential signal was digitized at 50 kHz using a Digidata 1440A A/D card, recorded with pClamp 10 software (both from Molecular Devices, Sunnyvale, CA, USA) and stored for later analysis. APs were recorded at pacing frequencies of 1 Hz, 2 Hz, 3.33 Hz, 4 Hz and 5 Hz in this respective order in the absence and in the presence of OM. APs were monitored at 1 Hz for 10 minutes before taking the records both in the DMSO-containing Tyrode solution and in the presence of 1 µM OM.

*Recording of cell length of isolated canine left ventricular cardiomyocytes*

Cell length (CL) was measured using a video-edge detector system (VED-105, Crescent Electronics, Sandy, Utah, USA) simultaneously with the AP recordings. The analogue signal was amplified (DC amplifier, Főnixcomp Ltd, Hungary), digitized at 240 Hz with a Digidata 1440A A/D converter and recorded with pClamp 10 software (both from Molecular Devices, Sunnyvale, CA, USA).

In some experiments, CL was measured without electrical stimulation. In these cases, CL was monitored in the DMSO-containing Tyrode solution for 10 minutes and then the cells were exposed to OM concentrations of 1nM - 10 µM for 10 minutes in each concentration.

The effect of OM on APD restitution was determined by pacing the myocyte at a steady-state cycle length of 500 ms for 20 consecutive beats and then a premature stimulus was given at a predefined diastolic interval (DI) as the 21^st^ beat [5]. The DI was set between 10-200 ms. On the APD restitution curve, the average APD_90_ (±SD) values of the 21^st^ AP were plotted against their respective DI. The procedure was then repeated on the same myocyte in the presence of 1 µM OM as well.

*Photometric detection of intracellular Ca^2+^ transients of isolated canine left ventricular cardiomyocytes*

Cardiomyocytes were loaded with 5 µM Fura-2 AM for 30 min at room temperature in a Pluronic F-127 containing Tyrode solution. 25 mg Pluronic F-127 was dissolved in 1 ml DMSO and this solvent was used to make a Fura-2 AM stock solution of 1 mM. After loading, the cells were washed twice with Tyrode solution, and were allowed to rest for 30 min at room temperature to de-esterify the dye, and then they were stored at 15 ºC before the experiments. Fluorescence was measured using an alternating dual beam excitation fluorescence photometry setup (RatioMaster; Photon Technology International, New Brunswick, NJ, USA) coupled to the inverted microscope. Fluorescence signals of Ca^2+^-bound and Ca^2+^-free Fura-2 dye were detected at excitation wavelengths of 340 nm (F_340_) and 380 nm (F_380_), respectively. Emitted photons were detected at 510 nm with an R1527P photomultiplier tube (Hamamatsu Photonics, Hamamatsu, Japan). This signal was digitized at 200 Hz using the FelixGX software (Photon Technology International, Edison, NJ, USA) and stored for offline analysis. Background fluorescence was measured by moving the cell out of the field of view, and it was subtracted from total fluorescence in order to obtain fluorescence originating exclusively from the preparation. Fluorescence ratio of F_340_/F_380_ was used to assess intracellular Ca^2+^ transients (CaT). The CaT was recorded in parallel with AP and CL.

*Statistics*

Results are expressed as mean ± SEM.

The normal distribution of the data was first tested by Shapiro-Wilk test upon statistical evaluation. In the cases of normal distribution, datasets with multiple groups were evaluated by analysis of variance (ANOVA) (ordinary in case of no pairing or Greenhouse-Geisser correction in the case of repeated measurements) and the multiple comparisons by Holm-Sidak test; while, datasets with two groups were evaluated by ordinary, parametric T-test.

In case of deviation from the normal distribution or low observation numbers (n<6), non-parametric tests were used. In these cases, Friedman's test for repeated measurements or Kruskal-Wallis test for independent measurements were used for multiple groups. Multiple comparisons were done by Dunn's test. For evaluating two groups Wilcoxon matched-pairs signed rank test (repeated measurements) or Kolgomorov-Smirnov test (unpaired values) was used.

A p value <0.05 was the criterion of significance.

Results in the supplementary information

The slope of the APD restitution curve is believed to be a collective measure of the recovery processes of all the ion channels and their interactions with voltage during the action potential (Qu et al., 2000). Occurrence of alternans is promoted by a steep APD restitution slope at short diastolic interval (DI).

In the n=7 canine left ventricular cardiomyocyte studied we found no significant difference between the APD_90_ values at any of the applied DI with the Student’s paired t-test, suggesting that OM causes alternans by a mechanism different than modulating ion channel recovery (Supplementary Figure 1).

References in the supplementary information

1. Horvath B, Szentandrassy N, Veress R, Almassy J, Magyar J, Banyasz T, Toth A, Papp Z, Nanasi PP (2017) Frequency-dependent effects of omecamtiv mecarbil on cell shortening of isolated canine ventricular cardiomyocytes. Naunyn Schmiedebergs Arch Pharmacol 390:1239-1246 doi:10.1007/s00210-017-1422-z

2. Kovacs A, Fulop GA, Kovacs A, Csipo T, Bodi B, Priksz D, Juhasz B, Beke L, Hendrik Z, Mehes G, Granzier HL, Edes I, Fagyas M, Papp Z, Barta J, Toth A (2016) Renin overexpression leads to increased titin-based stiffness contributing to diastolic dysfunction in hypertensive mRen2 rats. Am J Physiol Heart Circ Physiol 310:H1671-1682 doi:10.1152/ajpheart.00842.2015

3. Magyar J, Szentandrassy N, Banyasz T, Kecskemeti V, Nanasi PP (2004) Effects of norfluoxetine on the action potential and transmembrane ion currents in canine ventricular cardiomyocytes. Naunyn Schmiedebergs Arch Pharmacol 370:203-210 doi:10.1007/s00210-004-0954-1

4. Nagy L, Kovacs A, Bodi B, Pasztor ET, Fulop GA, Toth A, Edes I, Papp Z (2015) The novel cardiac myosin activator omecamtiv mecarbil increases the calcium sensitivity of force production in isolated cardiomyocytes and skeletal muscle fibres of the rat. Br J Pharmacol 172:4506-4518 doi:10.1111/bph.13235

5. Qu Z, Xie F, Garfinkel A, Weiss JN (2000) Origins of spiral wave meander and breakup in a two-dimensional cardiac tissue model. Ann Biomed Eng 28:755-771 doi:10.1114/1.1289474

6. Radovits T, Olah A, Lux A, Nemeth BT, Hidi L, Birtalan E, Kellermayer D, Matyas C, Szabo G, Merkely B (2013) Rat model of exercise-induced cardiac hypertrophy: hemodynamic characterization using left ventricular pressure-volume analysis. Am J Physiol Heart Circ Physiol 305:H124-134 doi:10.1152/ajpheart.00108.2013
